# HE4 and CA-125 kinetics to predict outcome in patients with recurrent epithelial ovarian carcinoma: the META4 clinical trial

**DOI:** 10.3389/fonc.2023.1308630

**Published:** 2024-01-11

**Authors:** Michel Fabbro, Pierre-Jean Lamy, Célia Touraine, Anne Floquet, Isabelle Ray-Coquard, Caroline Mollevi

**Affiliations:** ^1^ Medical Oncology Department, Montpellier Cancer Institute (ICM), Univ. Montpellier, Montpellier, France; ^2^ Clinical Research Unit, Clinique BeauSoleil, Aesio, Montpellier, France; ^3^ Genomic Analyzes Institute, Imagenome, Inovie, Montpellier, France; ^4^ Institute Desbrest of Epidemiology and Public Health, University of Montpellier, Institut National de la Santé et de la Recherche Médicale (INSERM), Montpellier Cancer Institute (ICM), Montpellier, France; ^5^ Medical Oncology Department, Bergonie Institute, Bordeaux, France; ^6^ Centre Léon Bérard Department of Medicine & Centre de Recherche en Cancérologie de Lyon, Lyon Recherche Innovation Contre le Cancer (LYRICAN), Université Claude Bernard Lyon I, Lyon, France

**Keywords:** epithelial ovarian carcinoma, biomarkers kinetic, CA-125, HE4 epithelial ovarian carcinoma, HE4

## Abstract

HE4 and CA-125 are used for epithelial ovarian cancer (EOC) screening, diagnosis, and follow-up. Our objective was to study HE4 and CA-125 kinetics in patients treated for recurrent EOC. Serum samples were prospectively collected before the first chemotherapy cycle and every 3 months until disease progression. Data from 89/101 patients could be analyzed. At baseline, the median CA-125 and HE4 concentrations were 210 IU/L (7–10,310) and 184 pM (31–4,836). Among the 12 patients (13%) with normal CA-125 (<35 IU/L) concentration, eight had HE4 concentration ≥75 pM, and among the 16 patients with normal HE4 concentration (18%), 12 had increased CA-125 concentration. The median nadir concentrations were 31 IU/L (3–8,744) for CA-125 and 75 pM (20–4,836) for HE4. The median times to nadir were 14 (0–130) weeks for CA-125 and 12 (0–52) weeks for HE4. In multivariate analysis, CA-125 and HE4 nadir concentrations (<35 IU/L, HR 0.35, 95% CI: 0.17–0.72 and<75 pM, HR 0.40, 95% CI: 0.20–0.79) and time to CA-125 and HE4 nadir (>14 weeks, HR 0.37, 95% CI: 0.20–0.70 and >12 weeks, HR 0.43, 95% CI: 0.23–0.83) were prognostic factors of progression-free survival. More investigations on HE4 kinetics could help to better monitor patients with CA-125 concentration within normal values.

## 1 Introduction

Epithelial ovarian cancer (EOC) is diagnosed late (advanced disease) in 75% of patients, and therefore its prognosis is poor and the 5-year overall survival rate is approximately 20–25% (1). Peritoneal invasion is a very frequent recurrence site. Besides clinical status, tumor markers are used for EOC detection, diagnosis, disease monitoring, and prognosis prediction (2). Clinical imaging (CT, PET, and MRI) has a limited value for EOC screening, diagnosis, peritoneal invasion quantification, and treatment efficacy assessment (3, 4).

Cancer antigen 125 (CA-125) is a dynamic marker of ovarian cancer. Its decrease predicts ovarian cancer cell death and response to therapy, whereas its increase is often the first indication of disease recurrence. CA-125 is the only tumor marker currently used for EOC diagnosis and follow-up. Many guidelines on CA-125 use in EOC management have been published to help with treatment decision-making (5–7). Moreover, serial CA-125 testing is commonly used to detect EOC recurrence after surgery and adjuvant therapy (8). In a meta-analysis, elevated CA-125 values correlated with disease progression in 89% of patients (9). Therefore, after treatment end, recurrence monitoring includes CA-125 measurement (7, 10). According to the Gynecologic Cancer Inter-group criteria, during serial CA-125 measurements, disease progression is suspected when CA-125 concentration doubles the upper limit of the reference range in two occasions separated by at least 1 week (11, 12). As CA-125 concentration is increased in 90% of patients with advanced EOC at diagnosis, treatment response monitoring with this serum marker is generally part of the follow-up. CA-125 half-life represents a prognostic factor for recurrence after chemotherapy. Specifically, a half-life<20 days has been associated with better disease-free survival compared with a half-life >20 days (28 months vs. 19 months) (13, 14). In patients with EOC who receive chemotherapy but not primary debulking surgery at diagnosis, CA-125 concentration normalization after three chemotherapy cycles has been correlated with better survival (15), although the number of cycles of chemotherapy remains a point of debate (16, 17). These results were confirmed by Riedinger et al. who showed that CA-125 nadir and half-life during induction chemotherapy were independent predictors of recurrence (18). Recently, it has been shown that the CA-125 ELIMination rate constant K value, defined as the CA-125 clearance during the first 100 days of chemotherapy in retrospective studies, represents a good prognostic factor of subsequent platinum-resistant disease relapse, progression-free survival (PFS), and also overall survival (15, 19).

Human epididymis protein 4 (HE4) belongs to the family of whey acidic four-disulfide core proteins (20) that are expressed in the epididymis epithelium and play a role in sperm maturation. HE4 is also strongly secreted by EOC cells (21). This marker is not increased in benign ovarian pathologies, unlike CA-125 (22). Moreover, HE4 is elevated in 50% of EOC in which the CA-125 concentration is within the normal range. Therefore, it is a more specific and sensitive EOC marker than CA-125 (23). Previous studies showed HE4’s usefulness in combination with CA-125 for EOC diagnosis in women with a pelvic mass, and it is included in the Risk of Ovarian Malignancy Algorithm (24, 25). The HE4 and CA-125 combination displays increased sensitivity and specificity compared with CA-125 alone. A meta-analysis performed using data from more than 6,000 patients confirmed HE4’s sensitivity and specificity for EOC diagnosis (26). An elevated pre-operative HE4 concentration in patients with known EOC has been associated with shorter overall survival (27–29), and the HE4 levels correlate with chemoresistance (30). HE4’s role in EOC detection and diagnosis is well known, but it should be better studied in patients with ovarian cancer recurrence during chemotherapy (31, 32). It could be useful particularly in patients with tumors that do not express CA-125. Moreover, HE4 prognosis and predictive value should be compared with the information provided by CA-125. HE4 concentration should be analyzed also during the post-treatment follow-up to determine whether HE4 could be useful for recurrence detection.

Besides their concentration at diagnosis, CA-125 and HE4 kinetics, half-life, and nadir are relevant to predict the prognostic outcomes in primary EOC (22).

The aim of this study was to determine in patients treated for recurrent EOC the value of HE4 and/or CA-125 baseline concentrations and kinetics to predict the response to chemotherapy and the post-treatment prognosis. Our analysis showed that elevated baseline CA-125 and HE4 concentrations predicted a shorter PFS in patients with recurrent EOC. Moreover, CA-125 and HE4 nadir concentrations and the time to nadir were prognostic factors when included in the same model.

## 2 Materials and methods

### 2.1 Study design

META4 was a multicenter observational study carried out at three French Comprehensive Cancer Centers between September 2010 and September 2014 to evaluate HE4 and CA-125 kinetics in patients with recurrent EOC. The main objective of the study was to assess the prognostic value of HE4, compared to CA-125, for PFS. The secondary objective was to assess the kinetic parameters of both markers and their prognostic values.

This study (EudraCT 2010-A00152-37) was approved by the local ethics committee (CPP Sud Méditerranée). All patients provided a written informed consent before inclusion in the study. The study was performed in accordance with the Good Clinical Practice Requirements and the Helsinki Declaration.

### 2.2 Patients

The inclusion criteria were as follows: ≥18-year-old patients with fallopian tube, ovarian, or peritoneum EOC recurrence and programmed to receive at least three cycles of chemotherapy (first, second, or third recurrence). The main exclusion criteria were as follows: another cancer treated in the previous 5 years and number of chemotherapy lines >3.

Serum samples were prospectively collected before the first chemotherapy cycle, during treatment, and every 3 months until disease progression.

### 2.3 CA-125 and HE4 quantification

CA-125 and HE4 were quantified using immunoassays. HE4 in serum was measured with the commercial EIA method (Fujirebio Diagnostics, Malvern, PA, USA; www.fujirebio.com). This test is a solid-phase, non-competitive immunoassay based on the direct sandwich technique using two mouse monoclonal antibodies, 2H5 and 3D8, against two epitopes in the C-terminal WAP-type four-disulfide core (WFDC) domain of HE4. The CA-125 concentration in serum was measured by electrochemiluminescence using the CA-125 II cobas kit (Roche). The following standard cutoffs were considered: 35 IU/L for CA-125 and 75 pmol/L for HE4, as previously defined (16). The results of CA-125 and HE4 quantification were blinded and did not contribute to the therapeutic decision-making.

### 2.4 Endpoints and assessment

The primary endpoint was to study the prognostic value of the baseline HE4 and CA-125 serum concentrations.

The secondary endpoints were to study the HE4 and CA-125 kinetics in patients with a recurrent disease and who are receiving chemotherapy, namely: plasma concentration at baseline ( $C_{0}$ ), half-life ( $t_{1/2}$ ), time to normalization ( $t_{\text{norm}}$ ), plasma concentration at nadir ( $C_{\text{nadir}}$ ), time to nadir ( $t_{\text{nadir}}$ ), doubling time ( $t_{\text{d}}$ ), and time to exceed the clinical threshold ( $t_{\text{ex}}$ ). The kinetic parameters (definition and calculation method) are precisely defined in **Table 1**. Mono-compartmental models were performed, first, with $k_{1},$ the slope associated with the decrease of the logarithm of the marker between baseline and nadir, and then with $k_{2}$ , the slope associated with the increase of the logarithm of the marker after nadir. Linear regression was used to estimate $k_{1}$ and $k_{2}$ (in semi-logarithmic scale).

**Table 1:** Definition and calculation of kinetic parameters.

Notation	Definition	Calculation method
$C_{0}$	Plasma concentration at baseline	
$t_{1/2}$	Half-life Note: If the calculated half-life was higher than the time to nadir, it was not considered in the analysis (replaced by missing data).	Time required to observe a 50% decrease in the plasma concentration from baseline: $t_{1/2}=\frac{-\ln\left(2\right)}{k_{1}}$ (mono-compartmental model) with $k_{1}$ the slope associated with the decrease of the neperian logarithm of the marker between baseline and nadir. A linear regression (in semi-logarithmic scale) between baseline and nadir is used to estimate $k_{1}$ (and thus $t_{1/2}$ ).
$t_{norm}$	Time to normalization Notes: (i) It cannot be calculated if the baseline concentration is lower than the threshold; (ii) if the calculated time to normalization was higher than the time to nadir, it was not considered in the analysis (replaced by missing data).	Time required (from baseline to nadir) to observe a value below the clinical threshold $C_{s}\;$ (i.e. 35 IU/L for CA-125 and 75 pM for HE4): $t_{norm}=\frac{\ln\left(C_{s}\right)-\;\ln\left(C_{0}\right)}{k_{1}}$ (mono-compartmental model) with $k_{1}$ , the slope associated with the decrease of the neperian logarithm of the marker between baseline and nadir.
$C_{nadir}$	Plasma concentration at nadir	Lowest plasma concentration observed during treatment until progression (if progression occurs during treatment) or until 1 month ± 7 days (maximum 38 days) after the treatment end date (otherwise).
$t_{nadir}$	Time to nadir	Time from baseline to nadir.
$t_{d}$	Doubling time Note: If the calculated doubling time was higher than the time to progression or time to follow-up, it was not considered in the analysis (replaced by missing data).	Time required to observe a 100% increase in the plasma concentration at nadir (from nadir): $t_{d}=\frac{\ln\left(2\right)}{k_{2}}$ (mono-compartmental model) with $k_{2,}\;$ the slope associated with the increase of the neperian logarithm of the marker after nadir. An estimate of $k_{2}$ (and thus $t_{d}$ ) is obtained using a linear regression (in semi-logarithmic scale) between nadir and progression (if progression occurs) or nadir and the last value assessed (otherwise).
$t_{ex}$	Time to exceed the clinical threshold (from nadir) Notes: (i) It cannot be calculated if the concentration at nadir is higher than the threshold concentration; (ii) if the calculated time to exceed the clinical threshold was higher than the time to progression or time to follow-up, it was not considered in the analysis (replaced by missing data).	Time required (from nadir) to observe a value above the clinical threshold $C_{s}\;$ (i.e. 35 IU/L for CA-125 and 75 pM for HE4): $t_{elev}=\frac{\ln\left(C_{s}\right)-\ln\left(nadir\right)}{k_{2}}$ with $k_{2}$ , the slope associated with the increase of the neperian logarithm of the marker after nadir.

PFS was defined from inclusion to the date of the first documented progression or the date of death from any cause. Treatment efficacy was assessed every three cycles of chemotherapy by clinical examination and CT according to the RECIST 1.1 criteria (33). Patients with partial or complete response to chemotherapy were considered responders, whereas patients with progressive disease were considered non-responders. In responders, a follow-up visit was performed every 3 months.

### 2.5 Statistical analyses

Descriptive analyses were performed on the per-protocol population defined as all eligible patients with CA-125 and/or HE4 data to allow calculating the kinetic parameters before nadir (at least two assessments before nadir—including nadir—and with a decreasing slope: $\hat{k_{1}}<0$ ) and/or after nadir (at least two assessments after nadir—including nadir—and with an increasing slope: $\hat{k_{2}}>0$ ). Survival analyses were performed only on patients with high-grade serous carcinoma.

Categorical variables were reported as the number of observations (*N*) and the frequency (%) of each modality. Continuous variables were reported as median, minimum, and maximum.

The median follow-up was calculated using the Schemper and Smith method. PFS was estimated using the Kaplan–Meier method. Multivariate analyses were performed using Cox proportional hazards models. Variables with (univariate) *p*-values<0.05 were selected for multivariate analysis, and a backward covariate selection was performed. Hazard ratios (HR) were reported with 95% confidence intervals (CI). The two parameters “time to normalization” and “time to exceed the clinical threshold” were not included in the multivariate model due to the large number of missing values and because the analyses would have been performed on a specific subpopulation of patients. Three multivariate analyses were performed: with only the CA-125 kinetic parameters, with only the HE4 kinetic parameters, and with CA-125 and HE4 kinetic parameters and the patients’ clinical characteristics. The validity of the proportional hazard assumptions was verified using Schoenfeld residuals in the final models. The Harrell’s C-index (which corresponds to the percentage of concordance between prediction and outcome) was calculated to evaluate the predictive accuracy of the different models. All tests were two-sided, and *p*-values<0.05 were considered significant. Statistical analyses were performed with STATA 16.0 (StatCorp, College Station, TX, USA).

## 3 Results

### 3.1 Patients’ characteristics

**Figure 1:**
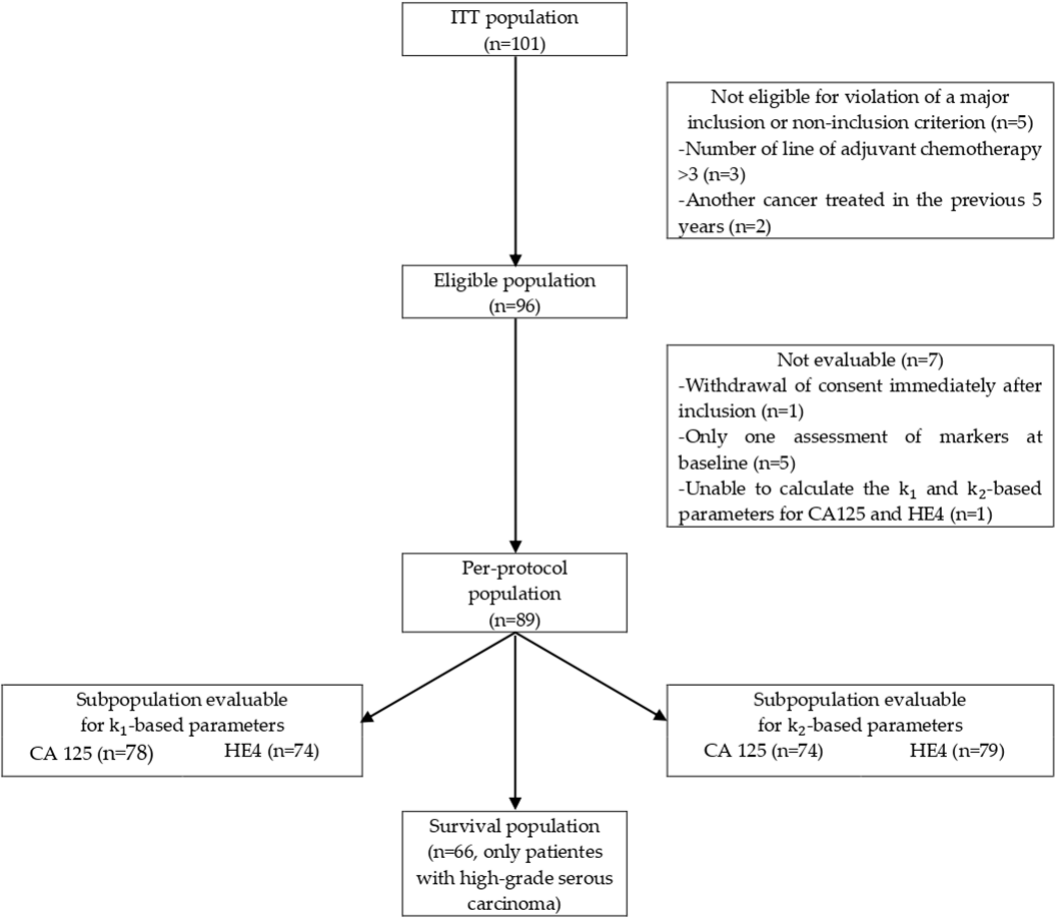
CONSORT flow chart.

From September 2010 to September 2014, 101 patients were included at the three centers (intention-to-treat population). Finally, 89 patients were included in the final analysis (per-protocol population). **Figure 1** summarizes the CONSORT flow chart.

The patient’s characteristics and treatment data are listed in **Tables 2**, **3**. At inclusion, the median age was 65 (34–83) years. The World Health Organization (WHO) performance status scores were 0, 1, and 2 in 40.5%, 48.3%, and 11.2% of patients, respectively; 88.5% of patients had high-grade carcinoma. The main histological sub-type was serous (87.5%).

**Table 2:** Patients’ demographic, clinical and histological characteristics.

	n=89	%
Center		
1 - ICM, Montpellier	72	80.9
2 - Institut Bergonié, Bordeaux	15	16.9
3 - Centre Léon Bérard, Lyon	2	2.3
Age		
Median (range)	65	(34-83)
WHO Performance status		
0	36	40.5
1	43	48.3
2/3	10	11.2
Residual disease		
No residual disease	35	35.3
≤ 1cm	37	19.6
> 1 cm	14	45.1
Missing	3	
FIGO stage		
I/II	6	6.8
III/IV	82	93.2
Missing	1	
Grade		
Low grade (1)	10	11.5
High grade (2 + 3)	77	88.5
Missing	2	
Histological type		
Serous	77	87.5
Endometrioïd	7	7.9
Undifferenciated	4	4.5
Clear Cell Carcinoma	1	0.1
CA-125 concentration (IU/L)		
Median (range)	210	7-10309
HE4 concentration (pM)		
Median (range)	184	31-4836
Creatinine clearance		
Median (range)	75	25-163
Missing	2	
Number of chemotherapy lines		
1	61	70.1
2	20	23.0
3	6	6.9
Missing	2	

**Table 3:** Treatment and efficacy.

	n=89	p%
Chemotherapy regimen		
Platinum-based	57	64
Carboplatin alone	4	4.4
Carboplatin in association (PLD, paclitaxel, gemcitabin)	53	59.6
Not Platinum-based	32	36
Paclitaxel weekly	26	29.3
PLD +/- trabectedin	5	5.6
Cyclophosphamide/bevacizumab	1	0.1
Best response		
CR	10	11.6
PR	39	45.7
SD	26	30.2
PD	11	12.8
Missing	3	
Reasons for stopping treatment		
Progression	34	38.2
Toxicity	3	3.4
Patient’s decision	2	2.3
Physician’s decision	37	41.6
Other	13	14.6

PLD, pegylated liposomal doxorubicin; CR, complete response; PR, partial response; SD, stable disease; PD, progressive disease.

At diagnosis, most patients (93.2%) had FIGO stage III or IV tumor, and 96.6% had undergone a surgery previously. Macroscopic residual disease was not detected in 35.3% of patients. This was the first, second, and third recurrence in 70%, 23%, and 7% of patients, respectively. Chemotherapy choice was left to the investigating physician according to the current recommendations on platinum-free interval before recurrence (shorter vs. longer than 6 months). Briefly, 64% of patients were treated with platinum-based chemotherapy [alone (4.4%) or associated with pegylated liposomal doxorubicin, paclitaxel, or gemcitabine (59.6%)], and 57% were platinum-sensitive. The other patients received mainly weekly paclitaxel (29.2%).

### 3.2 Biomarker kinetic parameters

At baseline (recurrence detection), the median CA-125 concentration was 210 IU/L (range, 7–10,310) and was ≥35 IU/L in 86.5% of patients (**Table 4**; **Supplementary Table S1**). The baseline HE4 median level was 184 pM (31–4,836), and was ≥75 pM in 82.0% of patients. The HE4 concentration was ≥75 pM in eight of the 12 patients with a normal CA-125 concentration (<35 IU/L) (**Supplementary Table S2**). The CA-125 concentration was increased in 12/16 patients with a normal HE4 concentration (<75 pM).

**Table 4:** Kinetic parameters.

	n=89	%
CA-125		
Concentration at baseline (IU/L)		
Median (min-max)	210 (7-10310)	
< 35	12	13.5
≥ 35	77	86.5
Half-life (weeks)		
Median (min-max)	6.5 (1.3-48.9)	
Missing	34	
Time to normalization (weeks)		
Median (min-max)	11.2 (2.8-20)	
Missing	57	
Nadir (IU/L)		
Median (min-max)	31 (3-8744)	
< 35	46	51.7
≥ 35	43	48.3
Time to nadir (weeks)		
Median (min-max)	14 (0-130)	
Doubling time (weeks)		
Median (min-max)	10.7 (1.1-39.9)	
Missing	34	
Time to exceed the clinical threshold (>35, weeks)		
Median (min-max)	34.4 (0.3-147)	
Missing	52	
HE4		
Concentration at baseline (pM)		
Median (min-max)	184 (31-4836)	
< 75	16	18.0
≥ 75	73	82.0
Half-life (weeks)		
Median (min-max)	8.5 (1.6-41.7)	
Missing	47	
Time to normalization (weeks)		
Median (min-max)	8 (1.8-23)	
Missing	60	
Nadir (pM)		
Median (min-max)	75 (21-4836)	
< 75	44	49.4
≥ 75	45	50.6
Time to nadir (weeks)		
Med (min-max)	12 (0-52)	
Doubling time (weeks)		
Median (min-max)	14.7 (2.1-67.3)	
Missing	36	
Time to exceed the clinical threshold (>75, weeks)		
Median (min-max)	21.7 (0.1-85.8)	
Missing	47	

The median CA-125 concentration at nadir was 31 IU/L (3–8,744) and was ≥35 IU/L in 48.3% of patients. The median HE4 concentration at nadir was 75 pM (21–4,836), and was ≥75 pM in 50.6% of patients. At nadir time, the HE4 concentration was ≥75 pM in 14 of the 46 patients with CA-125<35 IU/L (**Supplementary Table S2**).

**Figure 2:**
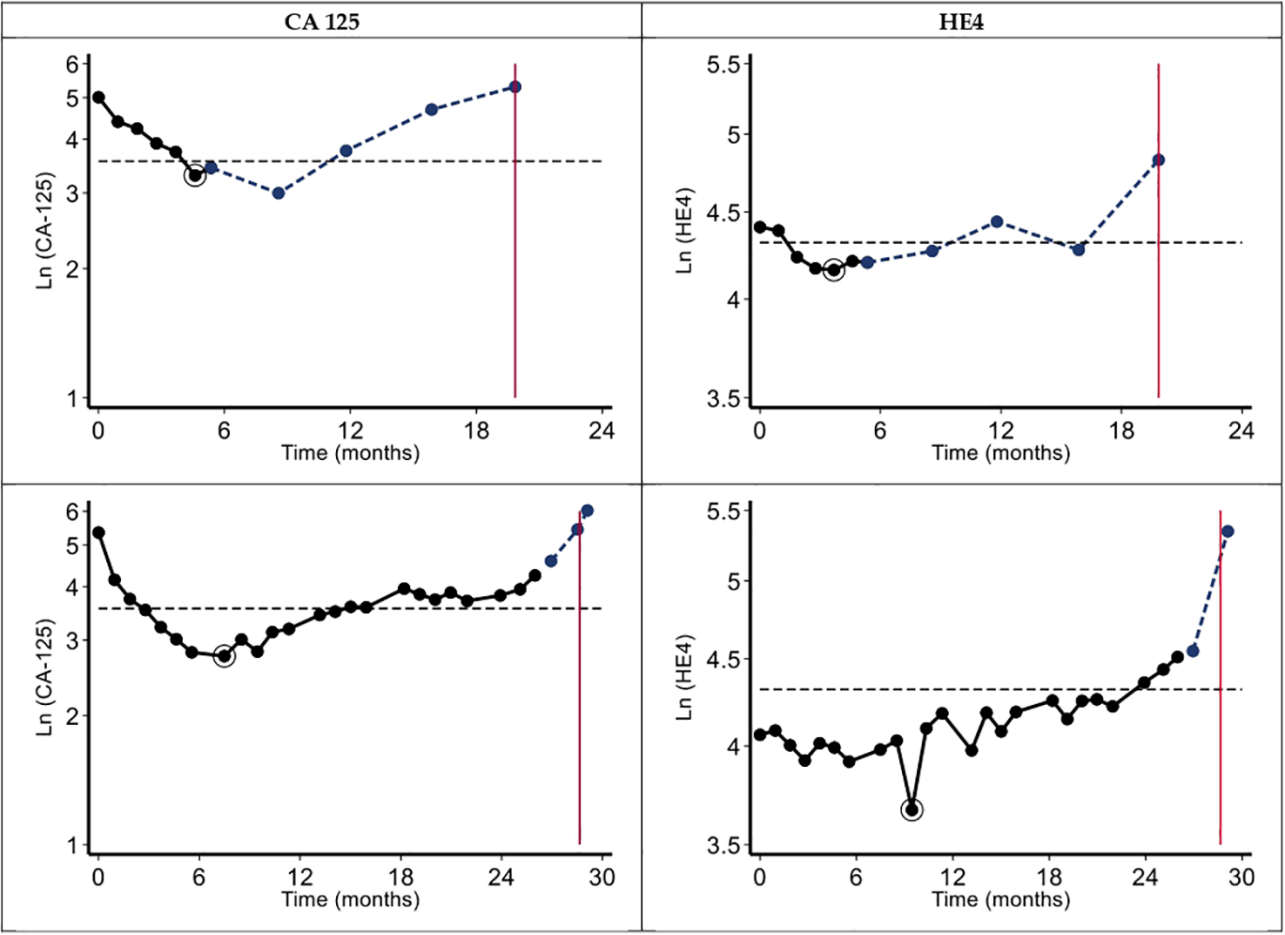
Trajectory of CA-125 and HE4. Black lines and dots represent the values of the markers observed under treatment, circled black dots show the value at nadir, blue lines and dots represent the values of the markers after the end of treatment, vertical red lines indicate the occurrence of progression, and horizontal dash lines indicate the threshold value.

The other kinetic parameters (half-time, time to normalization, time to nadir, doubling time, and time to exceed the clinical threshold) are described in **Table 4**. Two examples of CA-125 and HE4 kinetics (in semi-logarithmic scale) are shown in **Figure 2**.

Treatment response could be assessed in all patients, and 55% were considered as responders. The baseline CA-125 and HE4 concentrations were not significantly different in responders and non-responders: 197 IU/L (7–7,341) and 217 IU/L (25–10,309) for CA-125 (*p* = 0.21) and 176 pM (31–2,911) and 205 pM (46–4,836) for HE4 (*p* = 0.38), respectively. The half-life of both markers was not different in the responders and non-responders. Conversely, the CA-125 nadir concentration was significantly lower in the responders (16 IU/L, range: 3–796) than the non-responders (115 IU/L, range: 12–8,744, *p*< 0.001), and the time to nadir was longer in the responders (20 weeks, range: 4–130) than in the non-responders (8 weeks, range: 0–30, *p*< 0.001). Similar results were observed for HE4 (**Supplementary Table S1**).

### 3.3 Pronostic factors (univariate analysis)

The analysis was performed using data from 66 patients with high-grade carcinoma. Four patients were alive without progression at the study end, and the median follow-up was 12.1 months (95% CI: 9.3–12.6). The median PFS was 8.6 months (95% CI: 6.7–10.8).

The univariate analysis (**Table 5**) revealed that, among the clinical variables, only WHO performance status was a significant prognostic factor of PFS (0–1 vs. 2–3: HR 2.93, 95% CI: 1.34–6.39).

**Table 5:** Results of the univariate and multivariate analyses (n = 66).

	Univariate analysis				Multivariate analysis (model 1)				Multivariate analysis (model *2*)				Multivariate analysis (model 3)			
	n	HR	95% CI	*P*					n	HR	95% CI	*P*	n	HR	95% CI	*P*
Clinical characteristics																
**Age (years)**	66			0.386												
< 65		1														
≥ 65		1.25 [0.75, 2.08]														
**WHO Performance status**	66			0.016												
0-1		1														
2-3		2.93 [1.34; 6.39]														
**FIGO stage**	65			0.209												
I/II		1														
III/IV		2.90 [0.40; 21.13]														
**Residual disease**	64			0.224												
No residual disease		1														
≤ 1 cm or > 1 cm		1.39 [0.81, 2.40]														
CA-125																
**Concentration at baseline (IU/l)**	66			0.065												
< 35		1														
≥ 35		2.07 [0.89, 4.84]														
**Time to normalization (weeks)**	25			0.306												
< 11.2		1														
≥ 11.2		0.64 [0.27, 1.53]														
**Half-life (weeks)**	42			0.744												
< 6.5		1														
≥ 6.5		0.90 [0.47, 1.71]														
**Nadir (IU/l)**	66			<0.001	66			<0.001					66			0.004
< 35		1				1								1		
≥ 35		0.23 [0.13, 0.39]				0.19 [0.10, 0.35]								0.35 [0.17, 0.72]		
**Time to nadir (weeks)**	66			<0.001	66			<0.001					66			0.002
< 14		1				1								1		
≥ 14		0.32 [0.19, 0.54]				0.27 [0.15, 0.48]								0.37 [0.20, 0.70]		
**Doubling time (weeks)**	42			0.599												
< 10.7		1														
≥ 10.7		0.85 [0.46, 1.57]														
**Time to exceed the clinical threshold (>35, weeks)**	27			0.010												
< 34.4		1														
≥ 34.4		0.21 [0.08, 0.57]														
HE4																
**Concentration at baseline (pM)**	66			0.006												
< 75		1														
≥ 75		2.96 [1.24, 7.06]														
**Time to normalization (weeks)**	21			0.980												
< 8		1														
≥ 8		1.01 [0.39, 2.58]														
**Half-life (weeks)**	34			0.664												
< 8.5		1														
≥ 8.5		0.86 [0.43, 1.72]														
**Nadir (pM)**	66			<0.001					42			0.024	66			0.008
< 75		1								1				1		
≥ 75		0.27 [0.15, 0.48]								0.44 [0.21,0.92]				0.40 [0.20, 0.79]		
**Time to nadir (weeks)**	66			<0.001					42			<0.001	66			0.013
< 12		1								1				1		
≥ 12		0.27 [0.16, 0.45]								0.20 [0.10, 0.43]				0.43 [0.23, 0.83]		
**Doubling time (weeks)**	42			0.0141					42			0.004				
< 14.7		1								1						
≥ 14.7		0.44 [0.22, 0.86]								0.35 [0.17, 0.74]						
**Time to exceed the clinical threshold (>75, weeks)**	30			0.008												
< 21.7		1														
≥ 21.7		0.33 [0.15, 0.73]														

High baseline CA-125 and HE4 concentrations were associated with shorter PFS (HR 2.07, 95% CI: 0.89–4.84 and HR 2.96, 95% CI: 1.24–7.06, respectively). Conversely, low CA-125 and HE4 nadir concentrations were associated with longer PFS (HR 0.23, 95% CI: 0.13–0.39 and HR 0.27, 95% CI: 0.15–0.48, respectively). For CA-125, time to nadir ≥14 weeks and time to exceed the clinical threshold ≥34.4 weeks were strong prognostic factors of longer PFS (HR 0.32, 95% CI: 0.19–0.54 and HR 0.21, 95% CI: 0.08–0.57, respectively). Half-life and doubling time were not associated with PFS.

HE4 doubling time ≥14.7 weeks, time to nadir ≥12 weeks, and time to exceed the clinical threshold ≥21.7 weeks were strong prognostic factors of longer PFS (HR 0.44, 95% CI: 0.22–0.86, HR 0.27, 95% CI: 0.16–0.45; and HR 0.33, 95% CI: 0.15-0.73, respectively). As observed for CA-125, HE4 half-life was not a prognostic factor.

**Figure 3:**
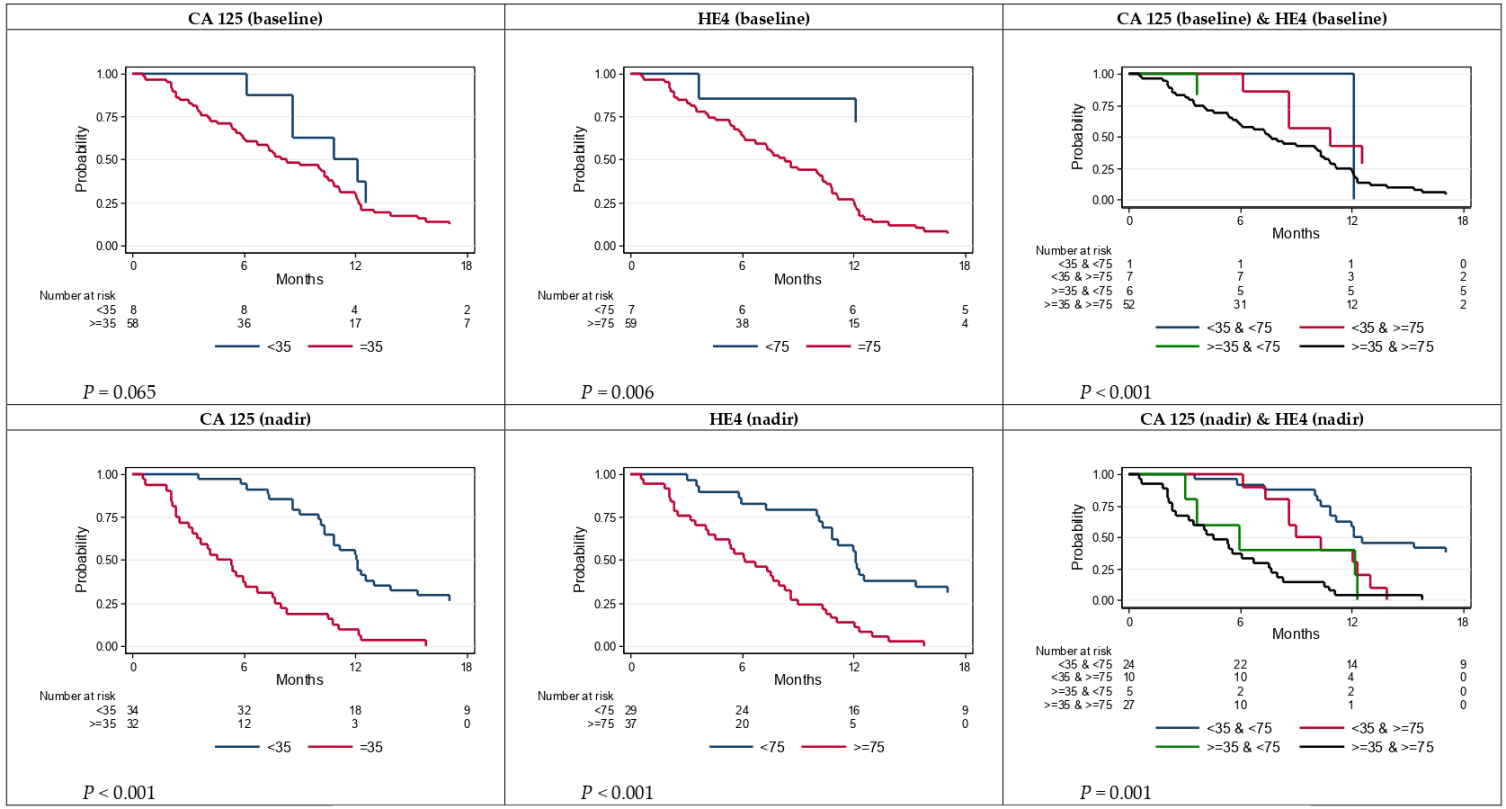
Progression-free survival according to CA-125 (baseline, nadir) and HE4 (baseline, nadir).

**Figure 4:**
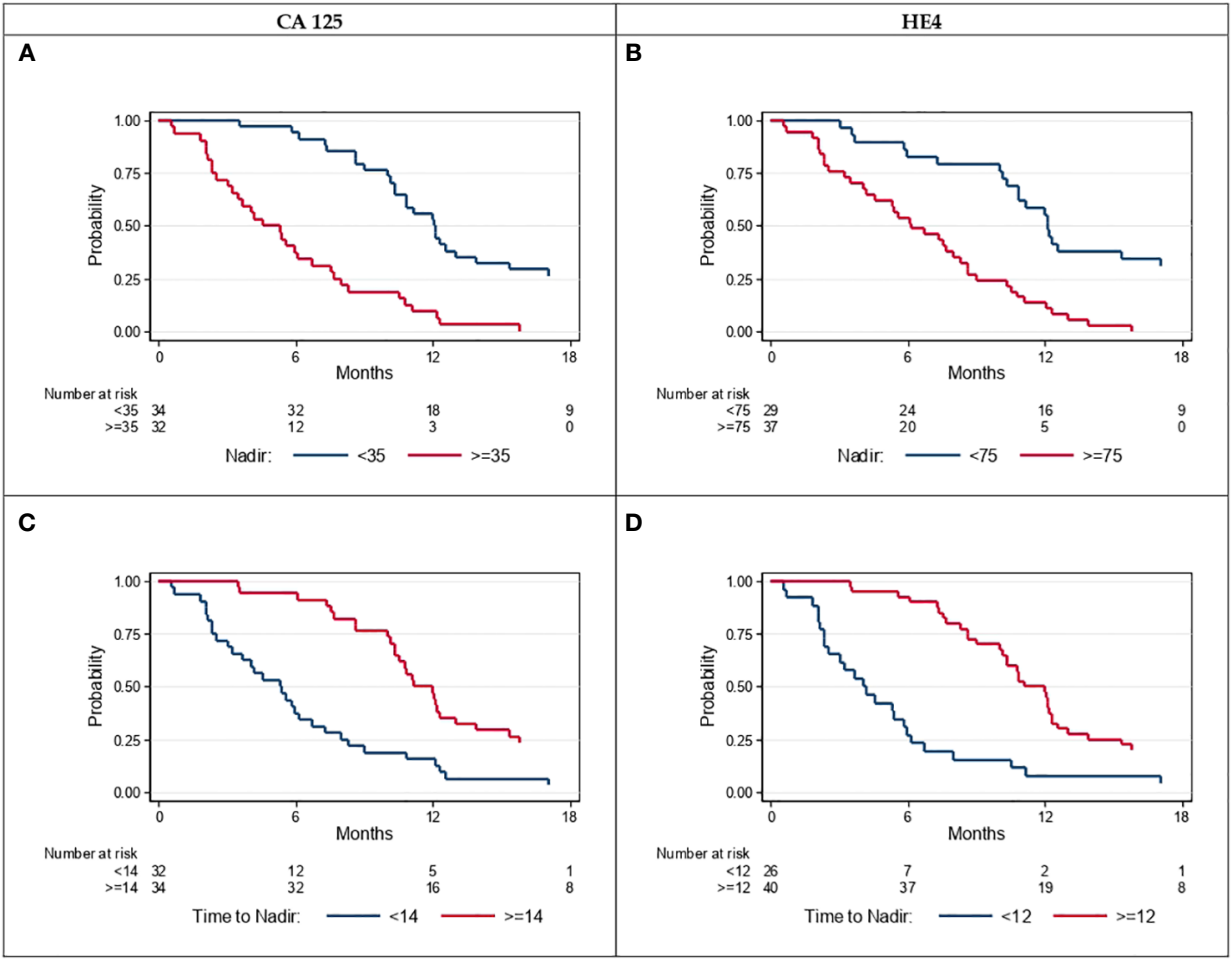
Progression-free survival according to CA-125 **(A)** threshold<35 vs. ≥35, **(C)** time to nadir<14 vs. ≥14 weeks and according to HE4 **(B)** threshold<75 vs. ≥75, **(D)** time to nadir<12 vs. ≥12 weeks.

The most significant prognostic parameters were baseline CA-125 and HE4 concentrations. **Figure 3** shows PFS in function of the baseline and nadir CA-125 and HE4 concentrations. PFS was always worse in patients with baseline CA-125 ≥ 35 IU/L and HE4 ≥ 75 pM (52/66; HR 3.65, 95% CI: 1.74–7.68 after grouping the other modalities) and nadir CA-125 ≥ 35 IU/L and HE4 ≥ 75 pM (27/66, HR 4.62, 95% CI: 2.62–8.13 after grouping the other modalities) (**Figure 4**).

### 3.4 Prognostic factors (multivariate analysis)

Three models were used from the multivariate analysis to identify the prognostic factors for PFS. The results are summarized in **Table 5**.

The multivariate analysis that included only CA-125 kinetic parameters led to a final model (model 1) with two significant factors: nadir concentration (*p*< 0.001) and time to nadir (weeks) (*p*< 0.001). Low CA-125 nadir concentration (<35 IU/L; HR 0.19, 95% CI: 0.10–0.35) and time to nadir ≥14 weeks (HR 0.27, 95% CI: 0.15–0.48) were independent favorable prognostic factors of PFS.

The multivariate analysis that included only HE4 kinetic parameters led to a final model (model 2) with three significant factors, namely: nadir concentration (*p* = 0.024), time to nadir (*p*< 0.001), and doubling time (*p* = 0.004). Low HE4 nadir concentration (≤75 pM; HR 0.44, 95% CI: 0.21–0.92), time to nadir ≥12 weeks (HR 0.20, 95% CI: 0.10–0.43), and doubling time ≥14.7 weeks (HR 0.35, 95% CI: 0.17–0.74) were independent favorable prognostic factors of PFS.

The multivariate analysis that included CA-125 and HE4 kinetic parameters and the patients’ clinical characteristics led to a final model (model 3) with four significant factors, namely: CA-125 nadir concentration (*p* = 0.004), time to CA-125 nadir (*p* = 0.002), HE4 nadir concentration (*p* = 0.008), and time to HE4 nadir (*p* = 0.013). Conversely, low CA-125 nadir concentration (<35 IU/L; HR 0.35, 95% CI: 0.17–0.72), time to CA-125 nadir ≥14 weeks (HR 0.37, 95% CI: 0.20–0.70), low HE4 nadir concentration (<75 pM; HR 0.40, 95% CI: 0.20–0.79), and time to HE4 nadir ≥12 weeks (HR 0.43; 95% CI: 0.23–0.83) were favorable prognostic factors.

For the three models, the proportional hazards assumption was not violated. According to the Harrell’s C-index, model 3 that included the kinetic parameters of both markers and the patients’ clinical characteristics was the best model, although the index values were similar for all models (0.75, 0.77, and 0.78 for model 1, 2, and 3, respectively).

## 4 Discussion

Most studies on the EOC biomarkers CA-125 and HE4 focused mainly on only one of them, although the main issue should be to determine what HE4 brings in addition to the well-known and universally used CA-125 marker. Moreover, many studies were carried out in neo-adjuvant settings where chemotherapy efficacy is tested in treatment-naive patients (34). On the other hand, the META 4 study assessed the prognostic values of both CA-125 and HE4 (baseline concentrations and kinetics) in patients with disease recurrence after previous chemotherapy cycles (i.e., not in adjuvant or neo-adjuvant settings). As described previously (REF), low (below the thresholds) CA-125 and HE4 nadir concentrations and long time to nadir were the main prognostic kinetic factors in addition to low grade histology.

At EOC recurrence time, prognostic factors are needed, for instance, to help with treatment decision-making, to monitor the treatment response, and to obtain information on survival. In our sample, the median baseline CA-125 concentration was 210 IU/L (7–10,310), similar to the 263 IU/L (5–52,000) concentration reported in a French multicenter study on 631 patients with EOC (18). Our study showed that both baseline CA-125 and HE4 concentrations have a high prognostic value, which is in agreement with previous studies. Elevated baseline CA-125 and HE4 concentrations predicted shorter PFS in patients with recurrent EOC: HR 2.07, 95% CI 0.89–4.84 and HR 2.96, 95% CI: 1.24–7.06, respectively (35). The same results are observed in neo-adjuvant treatment. Sensitivity to chemotherapy was predicted by both CA-125 and HE4, as described in previous studies (36–38).

The main result of our study was provided by the multi-variate analysis showing that both CA-125 and HE4 were independent prognostic factors for PFS, as indicated by the robust hazard ratios (0.35 and 0.40 for CA-125 and HE4 nadir concentrations, respectively). The nadir concentration and the time to nadir of CA-125 and HE4 were prognostic factors when included in the same model. This means that HE4 brings additional information to CA-125 nadir and time to nadir. This novel result could justify the use of both biomarkers. The role of HE4 in patients where CA-125 kinetic data do not correlate with disease progression warrants more investigation. In some cases (e.g., oligometastatic disease), early detection of progression could allow reductive surgery.

In EOC, surgical reduction of the tumor mass followed by platinum-based chemotherapy leads to complete remission in approximately 60% of patients and to CA-125 concentration normalization in 86% of patients receiving first-line chemotherapy (39–41). The relationship between chemotherapy efficacy, CA-125 concentration decrease, and survival has been strongly validated by several studies (9, 11, 42). In a recurrent disease, complete response and/or CA-125 normalization translates into a PFS improvement (43, 44). Our study confirmed the very strong prognostic value of CA-125 nadir concentration below the threshold (<35 IU/L) [HR = 0.23 and 0.35 in the uni- and multi-variate analyses, respectively, versus HR = 0.46 in previous studies (44, 45)]. Another study found that CA-125 nadir concentration after first-line treatment was associated with PFS, but not with overall survival (46).

Conversely, our study did not find any correlation of CA-125 or HE4 baseline concentration, half-time, and time to nadir with sensitivity to platinum-based chemotherapy, unlike what we observed for first-line chemotherapy (38). This highlights the fact that kinetic parameters represent more valuable information than a single quantification (even when abnormal) (42).

Surprisingly, long time to nadir (i.e., the slope between chemotherapy onset and the nadir) correlated with longer PFS. This suggests that the time won before reaching the nadir is time added to the date of recurrence. These results are not in accordance with what we previously observed during first-line chemotherapy, particularly in neo-adjuvant settings: faster CA-125 concentration decrease was associated with better treatment efficacy and significant PFS and overall survival improvement (15, 47). In recurrent EOC, reaching disease control, even partial, is more important than reaching rapidly the biomarker nadir. This time is currently prolonged by maintenance treatment, such as bevacizumab (43) and, more recently, PARP inhibitors (48–50).

This study presents some limitations, particularly the sample heterogeneity in terms of histology, although most patients (88.5%) had serous high-grade carcinoma. The second main limitation was the heterogeneity in platinum-free interval. Indeed 64% of patients received platinum-based chemotherapy, and 57% of them were platinum-sensitive. The third limitation was the treatment heterogeneity: platinum-based chemotherapy (alone or in association) and platinum-free treatment (36%). Lastly, the power of our study was limited by the small sample size.

## 5 Conclusions

Our study showed that HE4 kinetic information, in addition to CA-125 kinetic data, contributes to predict the prognosis (PFS) of patients with recurrent EOC treated by chemotherapy. More studies are needed especially in patients in whom the CA-125 concentration does not correlate with the disease course.

## Data availability statement

The raw data supporting the conclusions of this article will be made available by the authors, without undue reservation.

## Ethics statement

The studies involving humans were approved by EudraCT 2010-A00152-37 Local ethics committee (CPP Sud Méditerranée). The studies were conducted in accordance with the local legislation and institutional requirements. The participants provided their written informed consent to participate in this study.

## Author contributions

MF: Conceptualization, Formal analysis, Investigation, Supervision, Validation, Visualization, Writing – original draft, Writing – review & editing. P-JL: Conceptualization, Resources, Validation, Writing – original draft, Writing – review & editing. CT: Methodology, Writing – review & editing. AF: Investigation, Writing – review & editing. IR-C: Investigation, Writing – review & editing. CM: Conceptualization, Formal analysis, Methodology, Writing – review & editing.
