# Supplementary material for: HE4 and CA-125 kinetics to predict outcome in patients with recurrent epithelial ovarian carcinoma: the META4 clinical trial
Source: Front Oncol. 2024 Jan 11;13:1308630. doi: 10.3389/fonc.2023.1308630 (PMC10808592; doi:10.3389/fonc.2023.1308630)
Supplement: Supplementary file 1 [file Table_1.docx]

Supplementary Table 1. Kinetics parameters according to response to treatment.

|  |  | **Responders** | | **Non-responders** | | **Total** | | **P** |
| --- | --- | --- | --- | --- | --- | --- | --- | --- |
|  |  | **N=49** | **%** | **N=37** | **%** | **N=89** | **%** |  |
| **CA-125** | |  |  |  |  |  |  |  |
| **Concentration at baseline (IU/L)** | | | |  |  |  |  |  |
|  | Median (min-max) | 197 (7-7341) | | 217 (25-10309) | | 210 (7-10310) | | 0.209 |
|  | < 35 | 9 | 18.4 | 3 | 8.1 | 12 | 13.5 | 0.174 |
|  | ≥ 35 | 40 | 81.6 | 34 | 91.9 | 77 | 86.5 |  |
| **Half-life (weeks)** | | | |  |  |  |  |  |
|  | Median (min-max) | 6.5 (1.3-48.9) | | 6.4 (1.8-18.4) | | 6.5 (1.3-48.9) | | 0.900 |
|  | Missing | 10 |  | 22 |  | 34 |  |  |
| **Time to normalization (weeks)** | | | |  |  |  |  |  |
|  | Median (min-max) | 11.7 (2.8-20) | | 5.5 (2.8-12.6) | | 11.2 (2.8-20) | | 0.102 |
|  | Missing | 22 |  | 32 |  | 57 |  |  |
| **Nadir (IU/l)** | |  |  |  |  |  |  |  |
|  | Median (min-max) | 16 (3-796) | | 115 (12-8744) | | 31 (3-8744) | | <0.001 |
|  | < 35 | 38 | 77.6 | 8 | 21.6 | 46 | 51.7 | <0.001 |
|  | ≥ 35 | 11 | 22.4 | 29 | 78.4 | 43 | 48.3 |  |
| **Time to nadir (weeks)** | | | |  |  |  |  |  |
|  | Median (min-max) | 20 (4.0-130 | | 8.0 (0-30 | | 14 | 0-130 | <0.001 |
| **Doubling time (weeks)** | | | |  |  |  |  |  |
|  | Median (min-max) | 11.7 (1.1-39.9) | | 9.8 (1.6-28.9) | | 10.7 (1.1-39.9) | | 0.462 |
|  | Missing | 12 |  | 21 |  | 34 |  |  |
| **Time to exceed the clinical threshold**  **(>35 IU/L)weeks)** | | | |  |  |  |  |  |
|  | Median (min-max) | 36.3 (2.1-147) | | 8.9 (0.3-17.4) | | 34.4 (0.3-147) | | <0.001 |
|  | Missing | 20 |  | 30 |  | 52 |  |  |
| **HE4** | |  |  |  |  |  |  |  |
| **Concentration at baseline (pM)** | | | |  |  |  |  |  |
|  | Median (min-max) | 176 (31-2911) | | 205 (46-4836) | | 184 (31-4836) | | 0.385 |
|  | < 75 | 10 | 20.4 | 6 | 16.2 | 16 | 18.0 | 0.621 |
|  | ≥ 75 | 39 | 79.6 | 31 | 83.8 | 73 | 82.0 |  |
| **Half-life (weeks)** | | | |  |  |  |  |  |
|  | Median (min-max) | 10.3 (1.5-41.6) | | 6.7 (1.8-16.2) | | 8.5 (1.6-41.7) | | 0.064 |
|  | Missing | 19 |  | 26 |  | 47 |  |  |
| **Time to normalization (week)** | | | |  |  |  |  |  |
|  | Median (min-max) | 9.6 (2.1-23) | | 5.1 (1.8-16.6) | | 8 (1.8-23) | | 0.119 |
|  | Missing | 26 |  | 31 |  | 60 |  |  |
| **Nadir (pM)** | |  |  |  |  |  |  |  |
|  | Median (min-max) | 64 (21-430) | | 128 (35-4836) | | 75 (21-4836) | | <0.001 |
|  | < 75 | 32 | 65.3 | 12 | 32.4 | 44 | 49.4 | 0.003 |
|  | ≥ 75 | 17 | 34.7 | 25 | 67.6 | 45 | 50.6 |  |
| **Time to nadir (weeks)** | | | |  |  |  |  |  |
|  | Median (min-max) | 19.0 (0-52) | | 4.0 (0-27) | | 12.0 (0-52) | | <0.001 |
| **Doubling time (week)** | | | |  |  |  |  |  |
|  | Median (min-max) | 19 (5.9-67.3 | | 10.4 (2.1-33.1) | | 14.7 (2.1-67.3) | | 0.058 |
|  | Missing | 18 |  | 16 |  | 36 |  |  |
| **Time to exceed the clinical threshold**  **(>75 pM, weeks)** | | | |  |  |  |  |  |
|  | Median (min-max) | 25.4 (3.1-85.8 | | 8.9 (1.5-24.7) | | 21.7 (0.1-85.8) | | <0.001 |
|  | Missing | 20 |  | 25 |  | 47 |  |  |
