# Supplementary material for: HE4 and CA-125 kinetics to predict outcome in patients with recurrent epithelial ovarian carcinoma: the META4 clinical trial
Source: Front Oncol. 2024 Jan 11;13:1308630. doi: 10.3389/fonc.2023.1308630 (PMC10808592; doi:10.3389/fonc.2023.1308630)
Supplement: Supplementary file 2 [file Table_2.docx]

Supplementary Table 2. Baseline and nadir concentrations of CA-125 and HE4.

| **Concentration at baseline**  **n (%)** | | **CA-125** | | **Total** |
| --- | --- | --- | --- | --- |
|  |  | **< 35 IU/l** | $\boldsymbol{\geq}$ **35 IU/l** |  |
| **HE4** | **< 75 pM** | 4 (5%) | 12 (13%) | 16 (18%) |
|  | $\boldsymbol{\geq}$ **75 pM** | 8 (9%) | 65 (73%) | 73 (82%) |
| **Total** | | 12 (14%) | 77 (86%) | 89 (100%) |

| **Concentration at nadir**  **n (%)** | | **CA-125** | | **Total** |
| --- | --- | --- | --- | --- |
|  |  | **< 35 IU/l** | $\boldsymbol{\geq}$ **35 IU/l** |  |
| **HE4** | **< 75 pM** | 32 (36%) | 12 (13%) | 44 (49%) |
|  | $\boldsymbol{\geq}$ **75 pM** | 14 (16%) | 31 (35%) | 45 (51%) |
| **Total** | | 46 (52%) | 43 (48%) | 89 (100%) |
